# Variations of Soil Microbial Community Structures Beneath Broadleaved Forest Trees in Temperate and Subtropical Climate Zones

**DOI:** 10.3389/fmicb.2017.00200

**Published:** 2017-02-10

**Authors:** Sihang Yang, Yuguang Zhang, Jing Cong, Mengmeng Wang, Mengxin Zhao, Hui Lu, Changyi Xie, Caiyun Yang, Tong Yuan, Diqiang Li, Jizhong Zhou, Baohua Gu, Yunfeng Yang

**Affiliations:** ^1^Institute of Forestry Ecology, Environment and Protection, and the Key Laboratory of Forest Ecology and Environment of State Forestry Administration, the Chinese Academy of ForestryBeijing, China; ^2^State Key Joint Laboratory of Environment Simulation and Pollution Control, School of Environment, Tsinghua UniversityBeijing, China; ^3^School of Minerals Processing and Bioengineering, Central South UniversityChangsha, China; ^4^Department of Oncology, the Affiliated Hospital of Qingdao UniversityQingdao, China; ^5^Institute for Environmental Genomics and Department of Botany and Microbiology, University of OklahomaNorman, OK, USA; ^6^Earth Sciences Division, Lawrence Berkeley National LaboratoryBerkeley, CA, USA; ^7^Environmental Sciences Division, Oak Ridge National LaboratoryOak Ridge, TN, USA

**Keywords:** microbial community, GeoChip, high-throughput seuqencing, broadleaved forests, soil biogeochemical process

## Abstract

Global warming has shifted climate zones poleward or upward. However, understanding the responses and mechanism of microbial community structure and functions relevant to natural climate zone succession is challenged by the high complexity of microbial communities. Here, we examined soil microbial community in three broadleaved forests located in the Wulu Mountain (WLM, temperate climate), Funiu Mountain (FNM, at the border of temperate and subtropical climate zones), or Shennongjia Mountain (SNJ, subtropical climate). Although plant species richness decreased with latitudes, the microbial taxonomic α-diversity increased with latitudes, concomitant with increases in soil total and available nitrogen and phosphorus contents. Phylogenetic NRI (Net Relatedness Index) values increased from -0.718 in temperate zone (WLM) to 1.042 in subtropical zone (SNJ), showing a shift from over dispersion to clustering likely caused by environmental filtering such as low pH and nutrients. Similarly, taxonomy-based association networks of subtropical forest samples were larger and tighter, suggesting clustering. In contrast, functional α-diversity was similar among three forests, but functional gene networks of the FNM forest significantly (*P* < 0.050) differed from the others. A significant correlation (*R* = 0.616, *P* < 0.001) between taxonomic and functional β-diversity was observed only in the FNM forest, suggesting low functional redundancy at the border of climate zones. Using a strategy of space-for-time substitution, we predict that poleward climate range shift will lead to decreased microbial taxonomic α-diversities in broadleaved forest.

## Introduction

The ecological effects of global warming have widely been documented, with range shifts toward the poles at the rate of averagely 6.1 km per decade, and mean advancement of spring events by 2.3 days per decade (Camille and Gary, 2003). Forests are important ecosystems that support a large proportion of global biodiversity and store 45% of terrestrial carbon (Pan et al., 2011). In recent decades, substantial loss of carbon in forest soil has been observed, owing to positive feedbacks of forests to global warming via increasing fluxes of greenhouse gasses (Bonan, 2008; Heimann and Reichstein, 2008).

Broadleaved forests are major forest types in both temperate and subtropical climate zones (Wang et al., 2007). It is believed that (sub-)tropical forests are carbon neutral or carbon sinks, while temperate forests are usually carbon sources but can be turned into carbon sinks by reforestation and fire suppression (Pregitzer and Euskirchen, 2004). Subtle disturbance to the balance between respiration and photosynthesis can cause large changes in carbon pools from forests to atmosphere (Bonan, 2008), hence it is important to elucidate mechanisms of soil biogeochemical cycling and predict ecological consequences of forest extension northward by climate warming in the Northern hemisphere.

Soil microbial community is the major component of terrestrial biodiversity and primary driver of biogeochemical processes such as biomass decomposition and greenhouse gas emission (Crowther et al., 2014). Therefore, understanding the mechanisms in shaping community taxonomic and functional gene structure is essential for predicting soil functional capacity and ecosystem functions. However, it has been hampered by high complexity of microbial communities. This challenge has been alleviated by the rapid development of high-throughput, next-generation sequencing technologies. Sequencing of 16S rRNA gene amplicon is powerful in fine-tuning assessment of microbial taxonomic composition. Meanwhile, development of a functional gene microarray (GeoChip) using information from public sequence database has enabled quantitative, accurate and rapid detection of 100s of 1000s of functional genes (Tu et al., 2014). GeoChip 5.0 contains more than 50,000 oligonucleotide (50-mer) probes that can detect 393 functional genes from carbon, nitrogen, sulfur and phosphorus cycling, metal reduction and resistance, and organic contaminant degradation (He et al., 2007, 2010; Bracho et al., 2016).

In the current study, we collected soil samples located in the Wulu Mountain (WLM, 111°11.146′–111°11.235′ E, 36°33.399′–36°33.536′ N), Funiu Mountain (FNM, 111°48.150′–111°48.290′ E, 33°40.039′–33°40.086′ N) and Shengnongjia Mountain (SNJ, 110°21.570′–110°21.638′ E, 31°29.321′–31°29.462′ N). In climatology, FNM is located at the border of temperate and subtropical climate zones, while WLM is within a typical temperate climate zone and SNJ is within a subtropical climate zone.

Because of the substantial differences of the chemical and physical characteristics of broadleaved forest soils between temperate and subtropical climate zones, we hypothesize that niche-based mechanisms will explain soil community assembly through the selective power of plant or soil factors. However, it remains unclear whether selection of microbial taxonomic composition will differ from that of functional composition, given high frequency of horizontal gene transfer in natural environments (Thomas and Nielsen, 2005). Therefore, it is of great interest to understand under what condition and to what extent taxonomic compositions will be linked to functional compositions. Several previous studies have demonstrated that taxonomic compositions of microbial communities determine microbial functional capacities and processes (Waldrop and Firestone, 2006; Fierer et al., 2013), but other studies have showed disconnection between microbial community taxonomic compositions and its functions (Frossard et al., 2012; Crowther et al., 2014), which could be ascribed to functional redundancy of microbial communities, i.e., different taxonomic groups possess similar functions (Allison and Martiny, 2008). Therefore, it remains controversial whether and how to link microbial taxonomy to its functions.

## Materials and Methods

### Site Description and Soil Sampling

We collected soil samples in three natural broadleaved forests growing in mountain brown soils. Among them, WLM is located in Shanxi Province, typical of temperate monsoon climate. Ten sampling sites in WLM are located between 111°11.146′–111°11.235′ E, 36°33.399′–36°33.536′ N, and the elevation of 1814–1861 m above sea level (asl). The local climate is characterized by wet (precipitation of warmest quarter of over 300 mm), warm (mean temperature of warmest quarter of 17.5°C) weather in summer but dry and cold weather in winter. The plant community at WLM was dominated by arbor (*Quercus liaotungensis Koidz* and *Acer mono*), shrub (*Filipendula vulgaris, Lespedeza bicolor, Corylus mandshurica*, and *Acer stenolobum*) and grass (*Elymus dahuricus, Polygonatum odoratum, Stipa capillata, Carex, Epimedium, Thalictrum minus, Aster, Chrysanthemum chanetii*). FNM is located in Henan Province, the boundary of temperate and subtropical climate zones. Ten sampling sites are located between 111°48.150′–111°48.290′ E, 33°40.039′–33°40.086′ N, and the elevation of 1721–1791 m asl. The local climate is characterized by a warm (mean temperature of warmest quarter of 16.9°C), wet (precipitation of warmest quarter of over 450 mm) summer and mild winter. The plant community at FNM was dominated by arbor (*Pinus armandi, oriental white oak, Apricot linden, lindera obtusiloba*, and *Pinus tabuliformis*), shrub (*Rhododendron, Fargesia, Rosa multiflora, Smilax china*, and *Euonymus alatus*), and grass (*Oplismenus compositus, Carex, Elymus dahuricus*, and *Eriophorum*). The SNJ is located in Hubei Province and heavily influenced by subtropical monsoon. Ten sampling sites are located between 110°21.570′–110°21.638′ E, 31°29.321′–31°29.462′ N, and the elevation of 1725–1844 m asl. The local climate is characterized by warm (mean temperature of warmest quarter of 19.1°C), wet (precipitation of warmest quarter of over 500 mm) summer and very mild winter. The plant community at SNJ was dominated by arbor (*Ilex pernyi*, *Fagus engleriana*, *Q liena car acuteserata*, *Sorbus folgneri*, *Photinia beauverdiana, Lithocarpus, Cornus angustata*), shrub (*Fargesia robusta, Viburnum erosum, Wire handle carex, Viburnum dilatatum, Carpinus viminea, Lespedeza bicolor, Eurya groffii Merr, Symplocos botryantha, Smilax microphylla, Berberis julianae*) and grass (*Parathelypteris nipponica, Deyenxia langedorffii, Saussurea polycephala, Carex, Aster ageratoides, Tricyrtis macropoda*, and *Rubia cordifolia*).

The size of each of 10 sampling sites is 20 m × 20 m. At each site, 10–15 soil cores at the depth of 0–10 cm (A horizon) were taken, thoroughly mixed and sieved through 2 mm mesh to remove visible rocks and plant roots. Soil samples were preserved on ice when transported to laboratory, and then divided into two subsamples. Subsamples were stored at either 4°C for soil biogeochemical measurements, or -80°C for DNA extraction.

### Soil and Plant Factor Measurements

Plant factors were recorded *in situ* during soil sampling. Forests were divided into three canopies of trees, shrubs and arbors when counting plant species number and numbers of each species. Plant biomass was estimated by the diameter of breast height and height of trees. We used Shannon-Weaver index (H’) and Pielous evenness to calculate plant diversity and evenness, respectively. Soil geochemical factors, including soil pH, water content, organic carbon, total nitrogen, ammonium, nitrate, available nitrogen, total potassium, total sulfur, total phosphorus and available phosphorus, were measured as previously described (Ding et al., 2015a).

### DNA Extraction, Purification, and Quantification

We used freeze-grinding mechanical lysis to extract soil DNA, followed by purification twice with a 0.5% low melting point agarose gel and phenol-chloroform-butanol method (Yue et al., 2015). We assessed DNA quality by determining the ratios of 260 nm/280 nm and 260 nm/230 nm with a Nanodrop ND-1000 Spectrophotometer (NanoDrop Technologies, Inc., Wilmington, DE, USA). We quantified DNA amount via a PicoGreen method with a FLUO star Optima (BMG Labtech, Jena, Germany).

### Illumina Sequencing and Data Pre-processing

We amplified 16S rDNA genes with common primers (Forward primer, 515F, 5′-GTGCCAGCMGCCGCGGTAA-3′ and reverse primer, 806R, 5′-GGACTACHVGGGTWTCTAAT-3′) targeting v4 region of both bacteria and archaea, combined with adapter and barcode sequences. We then performed the two-step PCR amplification. The first round of PCR was carried out in triplicate in a 50 μl reaction using target-only forward and reverse primers, with 10 cycles for amplifications. Then products from the first round of PCR were purified with an Agencourt AMPure XP kit (Beckman Coulter, Beverly, MA, USA), eluted in 50 μl water and aliquoted into three 15 μl PCR tubes. The second round of PCR was carried out in triplicates in a 25 μl reaction, containing 2.5 μl of 10 × AccuPrime PCR bufferII (Invitrogen, Grand Island, NY, USA, including dNTPs), 0.4 μM of both forward and reverse primers, 5 μl of template DNA (2 ng/μl), 0.25 U of High Fidelity AccuPrime Taq Polymerase (Life Technologies) and 15 μl aliquot of PCR product purified from the first round. In this step, phasing primers with Illumina adapters, target primers, spacers and barcodes on the reverse primers were used. The amplifications were cycled for 20 times with the same program as the first round PCR. Quantification of PCR products were carried out with the PicoGreen method and confirmed by agarose gel electrophoresis. We diluted PCR products to 2 nM, then denatured DNA by mixing 10 μl of PCR products with 10 μl of 0.2 N fresh NaOH, followed by incubation for 5 min at room temperature. We then added 980 μl of chilled Illumina HT1 buffer to make a 20 pM library, which was further adjusted to 15 pM by adding HT1 buffer mixed with a PhiX DNA library. We loaded 600 μl of reaction mixture onto the MiSeq reagent cartridge (Illumina, San Diego, CA, USA) and conducted 2 bp × 250 bp paired-end sequencing.

We processed raw sequence data by a pipeline built on the Galaxy platform^1^ as recently described (Zhao et al., 2016). We first separated raw sequences into different samples based on barcodes associated with sequences, with only one mismatch permitted. We used Btrim to do quality trimming. Next, we used FLASH to merge forward and reverse reads into full length sequences, from which very short ones or ones that contained ambiguous bases were then removed. We also discarded chimeric sequences based on prediction by Uchime (usearch v5.2.3). Third, random re-sampling was achieved with 20,000 sequences per sample. Finally, we used UCLUST to cluster operational taxonomic units (OTUs) at the 97% similarity level, and removed the singletons. Taxonomic annotation of individual OTUs was conducted by RDP classifier with minimal 50% confidence estimates.

### GeoChip Hybridization and Data Pre-processing

We labeled 1 μg of purified genomic DNA with Cy3, then dried, rehydrated, and hybridized DNA with GeoChip 5.0 overnight as previously described (Ding et al., 2015a). After washing away unbound DNA, we scanned GeoChip with a NimbleGen MS 200 Microarray Scanner (Roche, Basel, Switzerland) at the level of 100% photomultiplier tube and 100% laser power. We considered probes with detected signal-to-noise ratio of less than 2.0 to be poor in quality and thus discarded them without further analyses. We also removed probes detected in no more than 3 out of 10 samples from each mountain to improve representation to the sites. We normalized signal intensity of each detected gene by dividing its signal intensity with total signal intensity of GeoChip. Finally, we transformed data to the natural logarithmic form.

### Statistical Analyses

We used the Shannon index (*H*’) to evaluate the α-diversity of microbial community. We used three non-parametric multivariate statistical tests of dissimilarity (adonis, MRPP, anosim), Detrended correspondence analysis (DCA) and hierarchical clustering analysis to examine differences of overall microbial community compositions. We used partial Mantel tests to unveil linkages between environmental factors and microbial community compositions. We also calculated the NRI and Nearest Taxon Index (NTI) to examine taxonomic traits conferring environmental tolerance or competitive abilities in three mountains. Specifically, NRI and NTI were conducted by package picante (v. 2.1-30) in R (v.3.1.1). All of those analyses were performed by functions in the R (v.3.1.1) Vegan package (v.1.15-1) as previously described (Liu et al., 2015), except for Analysis of variance by IBM SPSS statistic 19 and two-tailed unpaired *t*-tests by Microsoft Excel 2013 to determine the significance of the differences.

### Network Reconstruction Based on the RMT-Based Algorithm

We used the random matrix theory (RMT)-based algorithm to determine thresholds for Pearson correlation networks from both sequencing and GeoChip data (Ding et al., 2015b). We reconstructed networks of OTUs, carbon cycling genes and nitrogen cycling genes, which showed significant differences from random networks (Supplementary Table S4). We separated the networks into modules via the fast greedy modularity optimization. We calculated a number of topological properties of association networks, such as total nodes, total links, average degree, average connectivity, average clustering coefficient, average path distance, average length, density, modularity, transitivity, and connectedness.

## Results And Discussion

### Environmental Factors

A total of 24 environmental factors were measured, which could be classified as climatic, aboveground vegetation and soil geochemical factors (Supplementary Table S1). Since those three forests were geographically distant, there were significant (*P*< 0.050) differences among environmental factors, as shown by three non-parametric statistical tests of adonis, MRPP and anosim (**Table 1**). Particularly, precipitation decreased with latitudes, with roughly half of precipitation levels (annual precipitation, precipitation in the warmest season, and the wettest season) in the WLM forest compared to those in the SNJ forest (Supplementary Table S1). The mean air temperature (MAT) was also the lowest in the WLM forest and the highest in the SNJ forest. However, mean annual temperatures of the warmest and wettest seasons were the lowest in the FNM forest.

Species number of arbors, shrubs and grasses decreased with latitudes (Supplementary Table S1), which was in alignment with the general observation that microbial diversity is higher under warmer climate (Allen et al., 2002). The evolutionary speed hypothesis has suggested that biodiversity peaks at the equator due to higher mutation rates and shorter generation time of individuals under higher temperature (Mittelbach et al., 2007). However, microclimate could buffer under story plants responses to climate warming by denser canopies in forests, whose shading cools growing-season ground temperatures and thus attenuates the increase of warming-adapted species (De Frenne et al., 2013).

Soil geochemical factors varied considerably. Soil pH was neutral (6.98 ± 0.36) in the WLM forest but acidic in the FNM (5.01 ± 0.33) and SNJ forests (5.35 ± 0.45). Total organic carbon, total nitrogen, nitrate, available nitrogen, total phosphorus and available phosphorus, decreased with latitudes, suggesting that subtropical forests are poorer in nutrient conditions than temperate forests.

### Microbial Taxonomic Compositions

To analyze taxonomic compositions of microbial community, a total of 77,591 OTUs were detected in all of 30 soil samples, ranging from 5,887 to 10,595 OTUs per sample. The taxonomic α-diversity decreased with latitudes (Supplementary Table S2), and microbial OTU compositions differed significantly (*P*< 0.005) among three forests based on three non-parametric statistical tests of adonis, MRPP and anosim (**Table 1**). In accordance, DCA showed that samples of WLM, FNM and SNJ forests were well-separated from each other (Supplementary Figure S1A). Previous studies showed that microbial communities in forests with different aboveground vegetation were markedly different (Cong et al., 2015; Ding et al., 2015a). Nutrient quality or association between microbes and rhizospheres could also significantly change microbial communities (Weand et al., 2010; Urbanová et al., 2015).

**Table 1:** Pairwise dissimilarity test of environmental factors, 16S rRNA gene and functional gene data sets among any pairs of mountains.

Group	MRPP	MRPP	anosim	anosim	adonis	adonis
	Bray. Delta	Bray. *P*	Bray. *R*	Bray. *P*	Bray. *R*^2^	Bray. *P*
**Environmental factors**						
WLM vs. FNM	0.054	0.001	0.789	0.001	0.779	0.001
WLM vs. SNJ	0.054	0.002	0.818	0.001	0.809	0.001
FNM vs. SNJ	0.039	0.002	0.775	0.001	0.643	0.001
**OTUs**						
WLM vs. FNM	0.529	0.001	0.798	0.001	0.397	0.001
WLM vs. SNJ	0.540	0.001	0.790	0.001	0.418	0.001
FNM vs. SNJ	0.558	0.001	0.350	0.002	0.139	0.001
**Functional genes**						
WLM vs. FNM	0.081	0.001	0.456	0.001	0.258	0.001
WLM vs. SNJ	0.056	0.002	0.450	0.001	0.214	0.001
FNM vs. SNJ	0.083	0.002	0.031	0.001	0.197	0.004

The NRI was calculated to explore microbial phylogenetic relationship. NRI values were significantly (*P*< 0.050) different from zero in all of the three forests, with +0.519 for FNM samples and +1.042 for SNJ samples (**Table 2**). Those values suggested that FNM and SNJ samples were more phylogenetically related/clustered than expected by chance (i.e., phylogenetic clustering), probably resulting from habitat filtering of related species by environmental stress or competitive exclusion within community members (Goberna et al., 2014). Compared to FNM samples, higher NRI values in SNJ samples indicated a more phylogenetically clustered microbial assemblages, showing strong abiotic filtering in acidic, nutrient-poor subtropical forest soils (Smit et al., 2001). This is consistent with our hypothesis that soil community assembly is driven by niche-based mechanisms. In accordance, the *Alphaproteobacteria*-to-*Acidobacteria* ratio, a positive indicator of soil nutrient availability to microbes (Yergeau et al., 2012), was the lowest in the SNJ forest (Supplementary Table S1). In contrast, NRI value was -0.718 for WLM samples, suggesting that microbial assemblages were less phylogenetically related than expected by chance (i.e., phylogenetic over dispersion), which fitted the neutral model suggestive of demographic stochasticity and dispersal limitation as the shaping mechanism of microbial community taxonomy (Caruso et al., 2011).

**Table 2:** Metrics of phylogenetic compositions of soil microbial community.

	NRI	NTI
WLM	-0.718 ± 0.122	0.287 ± 0.218
FNM	0.519 ± 0.217	1.506 ± 0.427
SNJ	1.042 ± 0.106	2.332 ± 0.273

*NRI, Net Relatedness Index; NTI, Nearest Taxon Index.*

To identify differences of OTUs among three forests, we performed hierarchical clustering analysis with OTUs *in situ*, which showed that more than 4,000 OTUs were significantly different across the forests. For example, 9 *Proteobacteria* OTUs, which were among the most abundant OTUs, were substantially different in relative abundances across the forests (Supplementary Figure S2). Those *Proteobacteria* OTUs included five *Steroidobacter* (OTU_19792, OTU_112804, OTU_154253, OTU_370255 and OTU_239938), which were more abundant in the FNM and SNJ forests. Consistently, *Steroidobacter* grows on only a limited number of recalcitrant organic substrates and use nitrate or nitrite as the electron acceptor (Fahrbach et al., 2008). In addition, a *Proteobacteria* genera *Bradyrhizobium*, symbiotic nitrogen fixation bacteria forming nodules on soybeans (Kaneko et al., 2002), was the most abundant in the SNJ forest, which implicated that limited total and available nitrogen in soil could stimulate the microbial potential of nitrogen fixation.

### Microbial Functional Gene Structures

A total of 38,901 probes, representing more than 350 functional genes, had positive signals when hybridized with our soil sample DNA. We detected a total number of 878 bacterial taxa in GeoChip, of which 26.8% were also detected by MiSeq sequencing. Although the functional α-diversity was similar among three forests (Supplementary Table S2), dissimilarity tests of adonis, MRPP and anosim showed significant (*P*< 0.005) differences in functional gene structures (**Table 1**), which was verified by DCA (Supplementary Figure S1B).

Among all the carbon fixation genes, only *aclb* encoding ATP citrate lyase and *CODH* encoding carbon monoxide dehydrogenase were significantly (*P*< 0.050) higher in relative abundances in FNM samples (Supplementary Table S3). Other genes associated with carbon degradation were similar in relative abundances, lending support to the previous observation that decomposition rates of soil organic carbon in forest mineral soil were remarkably constant along a global-scale gradient in mean annual temperature (Giardina and Ryan, 2000). Since, the decomposition rate was limited by temperature only when the supply rate of soil organic carbon exceeded the consumption rate (Giardina and Ryan, 2000), constant decomposition rates can implicate that available organic carbon is restrained in forests. In contrast, no significant difference among three forests was observed for nitrogen cycling genes (Supplementary Table S3). However, high abundances of denitrification genes were detected across three forests, which provided supporting evidence for recent findings that microbial denitrification dominated nitrate losses from forest ecosystems (Fang et al., 2015).

### Association Networks

**FIGURE 1:**
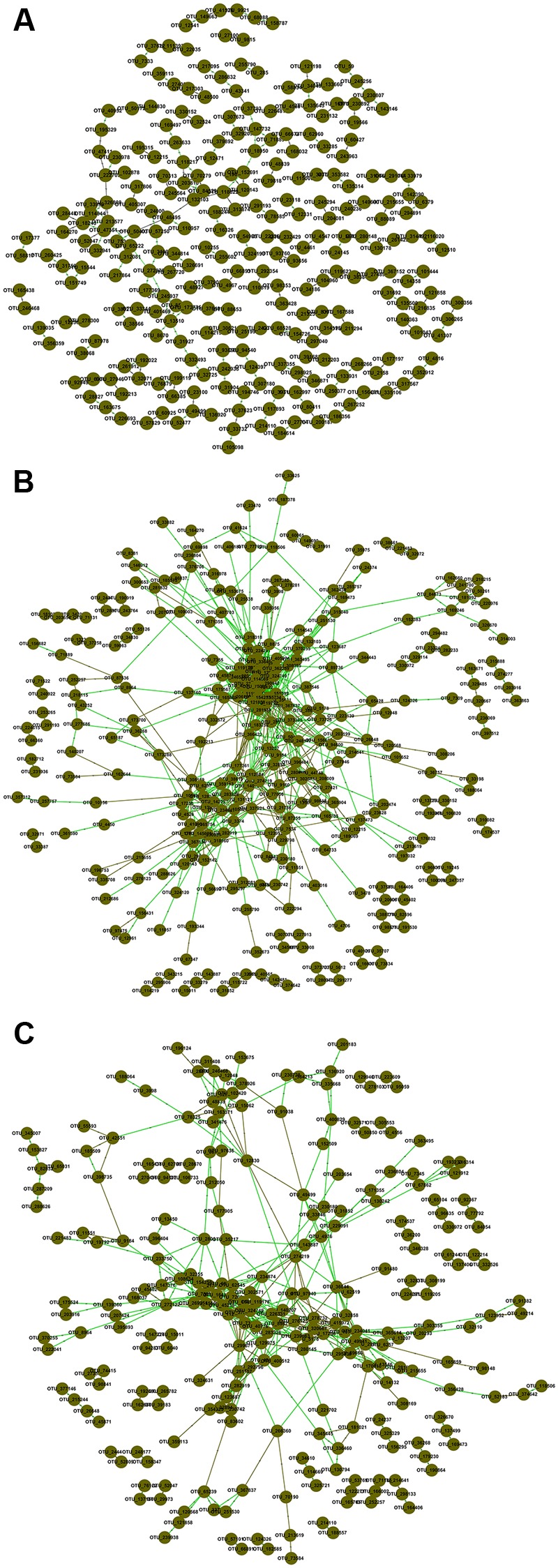
**Association networks of OTU data in (A)** WLM, **(B)** FNM, and **(C)** SNJ. The threshold was determined to be 0.920 based on the random matrix theory (RMT) algorithm. Nodes represent OTUs, green lines represent positive correlations, and brown lines represent negative correlations.

To explore possible ecological interactions within members of microbial communities, we generated correlation networks from OTUs data at the same threshold of 0.920. The resulting networks exhibited general topological features including scale free, small world and modularity (**Figure 1**; Supplementary Table S4). Topological properties of FNM and SNJ networks were similar, but distinct from those of the WLM network. The percentage of positive interactions, average connectivity, average path length, average clustering coefficient and transitivity were the lowest in the WLM network, but its modularity was the highest. In general, FNM and SNJ networks were highly connected, with densely connected nodes and longer average path length, which suggested slower response to environmental disturbance (Freedman and Zak, 2015).

The percentage of links between *Acidobacteria* and *Proteobacteria* OTUs was the highest in the SNJ network (31.85% of negative links and 25.24% of positive links), lower in the FNM network (18.26% of negative links and 22.15% of positive links), but the lowest in the WLM network (5.36% of negative links and 6.67% of positive links) (Supplementary Figure S3). The large percentage of links between *Acidobacteria* and *Proteobacteria* in the FNM and SNJ networks suggested their keystone role in shaping the microbial communities in the subtropical forests, where the soil was acidic and poor in nutrient.

**FIGURE 2:**
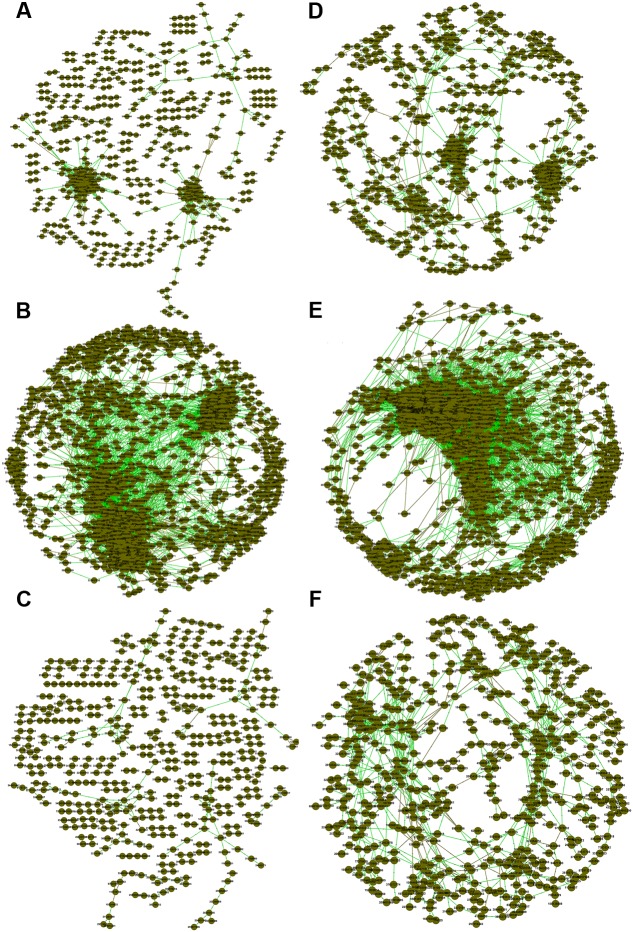
**Association networks of carbon cycling genes in (A)** WLM, **(B)** FNM, and **(C)** SNJ and nitrogen cycling genes in **(D)** WLM, **(E)** FNM, and **(F)** SNJ. The threshold was determined to be 0.970 for carbon cycling genes and 0.940 for nitrogen cycling genes, based on the RMT algorithm. Nodes represent gene probes, green lines represent positive correlations, and brown lines represent negative correlations.

We also generated correlation networks for carbon cycling genes (**Figure 2**). Interestingly, the functional gene network of FNM samples substantially differed from those of WLM and SNJ samples in terms of the number of nodes, percentage of positive interaction, average connectivity, average clustering coefficient and average path length (Supplementary Table S4). We also observed similar patterns of topological properties in nitrogen cycling genes (**Figure 2**; Supplementary Table S4).

### The Linkages between Microbial Community and Environmental Factors

Partial Mantel tests revealed that soil pH was among the strongest predictor for microbial taxonomic compositions across the forests (**Table 3**), which was consistent with previous studies (Fierer and Jackson, 2006). Although total carbon was rich in the FNM forest, low available phosphorus and nitrate can be limiting factors since partial Mantel tests revealed their significant (*P* < 0.050) linkages with microbial communities. At the functional gene level, soil nitrate and available nitrogen correlated with microbial functional gene structures in FNM samples. However, none of the environmental factors correlated with microbial functional gene structures in WLM or SNJ samples.

**Table 3:** The relationships of microbial community to environmental factors revealed by partial Mantel tests.

	WLM		FNM		SNJ	
	OTUs	Functional genes	OTUs	Functional genes	OTUs	Functional genes
		
	*R*_m_ (*P*)	*R*_m_ (*P*)	*R*_m_ (*P*)	*R*_m_ (*P*)	*R*_m_ (*P*)	*R*_m_ (*P*)
Arbor/shrub Shannon	-0.540 (0.999)	-0.120 (0.942)	0.480 (**0.038**)	0.100 (0.678)	-0.200 (0.930)	0.240 (0.728)
Arbor/shrub species number	-0.450 (0.999)	0.060 (0.617)	-0.040 (0.933)	0.060 (0.769)	0.080 (0.696)	0.230 (0.728)
Arbor/shrubPielous	-0.440 (0.999)	-0.170 (0.942)	0.250 (0.294)	0.000 (0.806)	-0.230 (0.963)	0.140 (0.728)
Arbor/shrub/grass Shannon	-0.500 (0.999)	-0.190 (0.942)	0.260 (0.257)	-0.130 (1.000)	-0.010 (0.696)	0.040 (0.728)
Arbor/shrub/grass species number	-0.440 (0.999)	-0.260 (0.965)	-0.250 (1.000)	-0.140 (1.000)	0.190 (0.542)	0.140 (0.728)
Arbor/shrub/grass Pielous	-0.530 (0.999)	-0.110 (0.942)	0.360 (0.197)	0.100 (0.605)	-0.010 (0.696)	0.080 (0.728)
Soil temperature 10 cm (°C)	-0.450 (0.999)	-0.160 (0.942)	-0.350 (1.000)	-0.270 (1.000)	-0.060 (0.698)	-0.080 (0.834)
pH	0.620 (**0.015**)	0.200 (0.420)	0.880 (**0.004**)	0.640 (**0.083**)	0.900 (**0.025**)	0.040 (0.728)
Water content (%)	0.510 (**0.039**)	0.330 (0.420)	0.610 (**0.007**)	0.500 (0.171)	0.050 (0.696)	0.170 (0.728)
Organic carbon (g/kg)	0.620 (**0.008**)	0.230 (0.420)	0.630 (**0.004**)	0.200 (0.408)	-0.000 (0.696)	-0.020 (0.728)
Total nitrogen (g/kg)	0.740 (**0.008**)	0.060 (0.617)	0.760 (**0.004**)	0.380 (0.140)	0.110 (0.679)	-0.010 (0.728)
NH_4_^+^ (mg/kg)	0.050 (0.630)	-0.200 (0.942)	-0.170 (1.000)	-0.260 (1.000)	-0.040 (0.696)	0.180 (0.728)
NO_3_^-^ (mg/kg)	0.460 (**0.029**)	0.080 (0.617)	0.830 (**0.004**)	0.640 (**0.083**)	-0.090 (0.698)	-0.210 (0.929)
Available nitrogen (mg/kg)	0.580 (**0.017**)	0.120 (0.617)	0.790 (**0.004**)	0.750 (**0.05**)	-0.030 (0.696)	0.020 (0.728)
Total potassium (g/kg)	0.030 (0.630)	-0.180 (0.942)	-0.190 (1.000)	-0.250 (1.000)	-0.050 (0.696)	-0.220 (0.929)
Total sulfur (g/kg)	0.620 (**0.008)**	0.080 (0.617)	-0.540 (1.000)	-0.320 (1.000)	0.340 (0.256)	0.010 (0.728)
Total phosphorus (g/kg)	0.700 (**0.013)**	0.070 (0.617)	-0.330 (1.000)	-0.350 (1.000)	-0.320 (0.983)	-0.020 (0.728)
Available phosphorus (mg/kg)	0.420 (**0.039**)	0.200 (0.420)	-0.060 (0.933)	-0.280 (1.000)	0.370 (0.242)	0.040 (0.728)

*P < 0.05 is shown in bold.*

### Correlations between Microbial Taxonomy and Functional Genes

**FIGURE 3:**
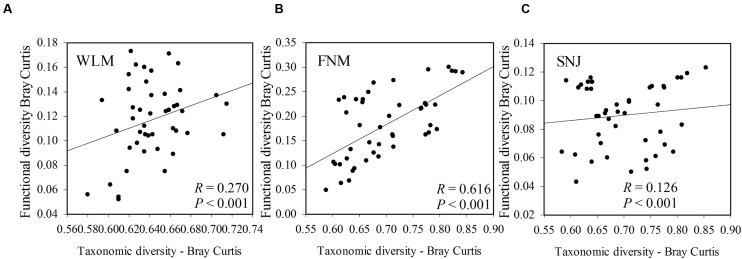
**The correlations between taxonomic and functional diversity in(A)** WLM, **(B)** FNM, and **(C)** SNJ samples. Bray–Curtis distances among samples were calculated and plotted to generate Pearson correlation values.

Functional redundancy is the degree to which different organisms execute similar function (Rosenfeld, 2002), or the capacity to remain functionally stabilized upon the loss of organisms (Nyström, 2006). A measure of functional redundancy was the correlation between taxonomic and functional gene diversities (Fierer et al., 2013). Correlations between the taxonomic and functional β-diversity were strong in FNM samples (*R*= 0.616, *P*< 0.001) but much weaker in WLM (*R*= 0.270, *P*< 0.001) and SNJ samples (*R*= 0.126, *P*< 0.001) (**Figure 3**), reflecting low functional redundancy in FNM. Further correlations between dissimilarity of abundant phylum and functional genes were also similar. One possible explanation is the shift from generalist species (possessing functional genes shared by many species, which is numerous in taxa such as *Alphaproteobacteria*) to specialist species (possessing specialized functional genes, which is numerous in taxa such as *Acidobacteria*), which results in an increase in functional diversity (Yergeau et al., 2012). We also observed strong and positive correlations (*R*= 0.871, *P*< 0.001) between the plant and microbial taxonomic α-diversity in FNM samples but weak correlations in WLM (*R*= 0.293, *P*< 0.001) and SNJ samples (*R*= 0.165, *P*< 0.001) (Supplementary Figure S4). Therefore, an alternative explanation is the influence of plant, which showed the highest α-diversity in FNM samples (Supplementary Table S1). At local scales, plant diversity, i.e., species and functional group richness, was previously shown to be the most important plant community property affecting soil microorganisms through root derived resources (Eisenhauer et al., 2011). Plant diversity could also stabilize belowground processes, possibly due to more consistent plant derived belowground inputs (Milcu et al., 2010). Specifically, plant exudate could release carbon substrate and stimulate microbial activity (Paterson, 2003; Mitchell et al., 2012; Pritchard et al., 2014). The excess of available carbon can lead to progressive available nitrogen limitation (Merckx et al., 1987). As a consequence, we found that available nitrogen showed the strongest linkages with both taxonomic and functional gene structures in FNM samples (*R* > 0.750, *P* < 0.050, **Table 3**), which could result in correlation between taxonomic and functional gene structures (**Figure 3**).

**FIGURE 4:**
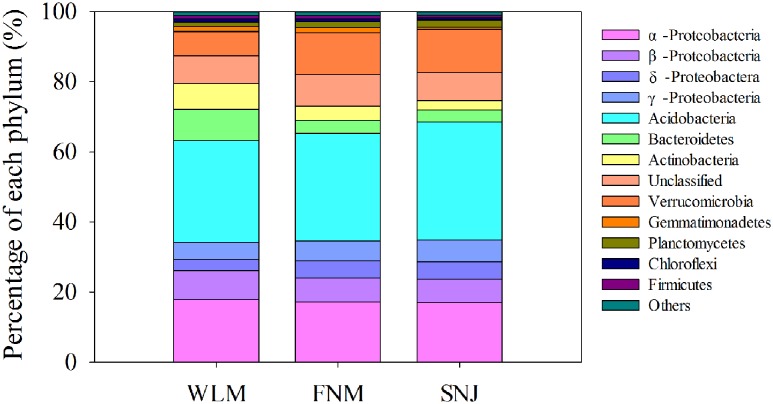
**Comparison of the taxonomic distribution of the most important phyla among the WLM, FNM, and SNJ forests**.

Low functional redundancy could lead to ecological consequences in system’s resistance to environmental changes. Notably, the FNM network of nitrogen cycling genes did not exhibit a modular structure (Supplementary Table S4; values < 0.4 suggest that the network is non-modular; Newman, 2006). *Verrucomicrobia*, a relatively slow-growing taxon thriving in environments with limited nutrients, showed the strongest correlations with functional genes (Supplementary Table S5). This phylum was reported to drive the biogeographical patterns in prairie soils, which were associated with strong shifts in carbon dynamics (Fierer et al., 2013). Specifically, *Verrucomicrobia* (6.9% of total OTUs at WLM, 11.9% of total OTUs at FNM and 12.3% of total OTUs at SNJ, **Figure 4** and Supplementary Figure S5) correlated with various genes associated with carbon degradation and nitrogen metabolism (Supplementary Table S6), verifying recent observations in *Verrucomicrobia* metabolisms (Fierer et al., 2013).

### Projection

Global climate warming has caused climate range shift toward poles (Camille and Gary, 2003). Using a strategy of space-for-time substitution, we predict that poleward shift of the subtropical climate will decrease total organic carbon, total and available nitrogen and phosphorus contents in FNM and WLM soils (Supplementary Table S1). This prediction falls into a much bigger picture predicted by simulations included in the IPCC fourth assessment report that nitrogen is a primary limiting nutrient in subtropical and tropical terrestrial ecosystems, and the capacity of the terrestrial biosphere to store anthropogenic carbon emissions is diminishing over the 20th and 21st centuries as climate changes (Menon et al., 2007).

Based on our analyses of microbial taxonomic and functional traits, we found that soil microbial community in subtropical forests showed lower taxonomic diversities than temperate forests and shifted from phylogenetic over dispersion to clustering, probably as a consequence of environmental filtering of soil acidification and loss of nutrients. However, considering the importance of fungi and Archaea in mediating underground processes in forests (Stursova et al., 2014), it needs further investigation into soil fungal and Archaeal community to better understand the responses of forests to environmental changes. Given strong metabolic flexibility and adaptability of soil microbial communities (Bardgett and Van Der Putten, 2014), a time-series period experiment would be necessary to examine whether warming will exert permanent effects on microbial communities and other components in biosphere (Lau and Lennon, 2011).

## Data Accession

High-throughput sequencing can be found at NCBI database by the accession number SRP095499 (https://www.ncbi.nlm.nih.gov/sra/?term=SRP095499). GeoChip data can be found at NCBI databases by the accession number GSE92233 (http://www.ncbi.nlm.nih.gov/geo/query/acc.cgi?acc=GSE92233).

## Author Contributions

SY and YZ analyzed data and wrote paper and contributed equally to this work. YZ, JZ, and YY designed the experiment and supervised all work. CY and TY contributed to Illumina sequencing and GeoChip experiments. HL, JC, and DL collected sample and measured environmental parameters. MW, MZ, CX, and BG contributed to final revision of the manuscript.

## Conflict of InterestStatement

The authors declare that the research was conducted in the absence of any commercial or financial relationships that could be construed as a potential conflict of interest.

## Footnotes

1 http://zhoulab5.rccc.ou.edu:8080/
