# Supplementary material for: Variations of Soil Microbial Community Structures Beneath Broadleaved Forest Trees in Temperate and Subtropical Climate Zones
Source: Front Microbiol. 2017 Feb 10;8:200. doi: 10.3389/fmicb.2017.00200 (PMC5300970; doi:10.3389/fmicb.2017.00200)
Supplement: Supplementary file 1 [file Data_Sheet_1.DOCX]

Supplementary materials

Analyses of microbial community taxonomic and functional traits in broadleaved forest soils reveal potential consequences of range shifts by warming

Sihang Yang^1,2,†^, Yuguang Zhang^1,†^, Jing Cong^1,3,4^, Mengmeng Wang^2^, Mengxin Zhao^2^, Hui Lu^1^, Changyi Xie^2^, Caiyun Yang^5^, Tong Yuan^5^, Diqiang Li^1^, Jizhong Zhou^2,5,6^ , Baohua Gu^7^ and Yunfeng Yang^2,*^

^†^These authors contributed equally to this work.

^*^Correspondence: [yangyf@tsinghua.edu.cn](mailto:yangyf@tsinghua.edu.cn); Phone: +86-010-62784692; Fax: +86-010-(+86) 10-62785687;

## Supplementary data

## Table list:

**Table S1** Environmental factors in the WLM, FNM and SNJ forests.

**Table S2** α-diversities of sequencing and GeoChip data sets.

**Table S3** Average relative abundances of overall carbon functional groups, carbon fixation genes, methane cycling genes, carbon degradation genes, and nitrogen cycling genes.

**Table S4** Topology properties of networks and random networks. To generate reliable correlations, we removed OTUs or functional genes detected in less than nine out of ten replicates collected in each mountain. As quantitative measurement of abundant genes was more accurate than those of rare genes, we used the most abundant 3,000 genes associated with carbon cycling for network reconstruction.

**Table S5** R values of correlations between phyla and functional dissimilarities in three forests.

**Table S6** R values of correlations between functional gene categories and Verrucomicrobia taxonomic dissimilarities in three forests.

**Table S1** Environmental factors in the WLM, FNM and SNJ forests.

| Environmental factors (unit) | WLM^a^ | FNM | SNJ |
| --- | --- | --- | --- |
| **Aboveground vegetation** | |  |  |
| Arbor and shrub Shannon | 1.327b^b^ | 2.161a | 2.139a |
| Arbor and shrub species number | 7.900c | 17.600b | 28.500a |
| Arbor and shrub Pielous | 0.638b | 0.648b | 0.758a |
| Arbor, shrub and grass Shannon | 2.170a | 2.491a | 2.240a |
| Arbor, shrub and grass species number | 20.800c | 28.800b | 37.300a |
| Arbor, shrub and grass Pielous | 0.623b | 0.715ab | 0.748a |
| **Climate-related factors** | |  |  |
| Soil Temperature_10cm (^o^C) | 0.715ab | 0.748a | 0.623b |
| Mean Air Temperature (^o^C) | 5.840c | 6.400b | 9.500a |
| Mean Temperature Warmest quarter (^o^C) | 17.540b | 16.900c | 19.100a |
| Mean Temperature Wettest quarter (^o^C) | 16.040b | 15.700c | 18.300a |
| Annual Precipitation (mm) | 558.800c | 976.000b | 1234.000a |
| Precipitation Warmest quarter (mm) | 312.400c | 467.000b | 520.000a |
| Precipitation Wettest quarter (mm) | 329.800c | 484.000b | 532.000a |
| **Soil geochemical factors** | |  |  |
| pH | 6.98a | 5.01b | 5.35b |
| Water content (%) | 30.003b | 27.772b | 48.654a |
| Organic C (g kg^-1^) | 55.277a | 55.365a | 29.138b |
| Total N (g kg-1) | 4.350a | 3.883a | 1.924b |
| NH_4_^+^ (mg kg^-1^) | 12.642c | 30.820a | 19.539b |
| NO_3_^-^ (mg kg^-1^) | 34.040a | 8.207b | 9.438b |
| Available N (mg kg^-1^) | 322.608a | 269.007a | 183.532b |
| Total K (g kg^-1^) | 2.754b | 2.002c | 3.837a |
| Total S (g kg^-1^) | 0.682a | 0.643a | 0.545a |
| Total P (g kg^-1^) | 0.526a | 0.459a | 0.231b |
| Available P (mg kg^-1^) | 35.601a | 7.060b | 3.300b |
| α-Proterbacteria to Acidobacteria ratio | 0.616a | 0.560b | 0.505c |

Abbreviations: C, carbon; N, nitrogen; K, potassium; S, sulfur; P, phosphorus.

^a^WLM: soil located in Shanxi Province, temperate zone; FNM: soil located in Henan Province, the boundary between temperate and subtropical zones; SNJ: soil located in Hubei Province, subtropical zone.

^b^Values in the table are the mean values of ten replicates. Letters behind the values indicate significant differences (*P* < 0.050) among different sites, as determined by one-way analysis of variance in the SPSS. If one site shares a letter with another site, the difference between these two sites is insignificant (*P* > 0.050).

**Table S2** α-diversities of sequencing and GeoChip data sets.

|  | Richness | Shannon diversity | Pielous's evenness |
| --- | --- | --- | --- |
| **OTUs** |  |  |  |
| WLM^a^ | 8782a^b^ | 8.215a | 0.905a |
| FNM | 8818a | 8.167ab | 0.899ab |
| SNJ | 8050a | 7.895b | 0.879b |
| **Functional genes** | |  |  |
| WLM | 22140.5a | 9.966a | 0.996a |
| FNM | 22600.5a | 9.983a | 0.996a |
| SNJ | 22653.9a | 9.991a | 0.996a |

^a^WLM: soil located in Shanxi Province, temperate zone; FNM: soil located in Henan Province, the boundary between temperate and subtropical zones; SNJ: soil located in Hubei Province, subtropical zone.

^b^Values in the table are the mean values of ten replicates. Letters behind the values indicate significant differences (*P* < 0.050) among different sites, as determined by one-way analysis of variance in the SPSS. If one site shares a letter with another site, the difference between these two sites is insignificant (*P* > 0.050).

**Table S3** Average relative abundances of overall carbon functional groups, carbon fixation genes, methane cycling genes, carbon degradation genes, and nitrogen cycling genes.

|  | **WLM^a^** | **FNM** | **SNJ** |
| --- | --- | --- | --- |
| Total carbon | **6.706±0.034c^b^** | **6.737±0.065b** | **6.844±0.023a** |
| Carbon degradation | **6.685±0.034c** | **6.734±0.064b** | **6.831±0.023a** |
| Carbon fixation | **6.826±0.033b** | **6.81±0.064b** | **6.946±0.025a** |
| Methane | 5.745±0.046 | 5.717±0.096 | 5.883±0.026 |
| Starch degradation | **6.775±0.031b** | **6.849±0.063b** | **6.943±0.023a** |
| Hemicellulose degradation | 6.776±0.033 | 6.83±0.059 | 6.904±0.022 |
| Cellulose degradation | 6.509±0.036 | 6.503±0.067 | 6.657±0.024 |
| Chitin degradation | 6.435±0.038 | 6.45±0.07 | 6.556±0.023 |
| Lignin degradation | 6.492±0.041 | 6.527±0.078 | 6.655±0.033 |
| **Carbon fixation** | |  |  |
| aclB | **6.217±0.013b** | **7.351±0.255a** | **6.137±0.014b** |
| CODH | **5.456±0.116b** | **6.277±0.23a** | **5.815±0.08b** |
| rubisco | 6.781±0.129 | 6.451±0.213 | 6.604±0.078 |
| **Methane** |  |  |  |
| mmoX | 5.826±0.21 | 5.623±0.267 | 5.679±0.141 |
| pmoA | 5.717±0.13 | 5.749±0.324 | 5.954±0.068 |
| mcrA | 5.887±0.162 | 5.97±0.27 | 6.013±0.098 |
| **Carbon degradation** | | |  |
| amyA | 6.852±0.096 | 6.921±0.2 | 7.021±0.069 |
| amyx | **5.128±0.295b** | **6.894±0.222a** | **6.895±0.309a** |
| apu | **4.455±0.017b** | **6.701±0.446a** | **4.796±0.44b** |
| cda | 6.033±0.13 | 6.068±0.193 | 6.099±0.103 |
| glucoamylase | 6.714±0.106 | 6.841±0.192 | 6.938±0.099 |
| pula | 6.037±0.123 | 6.044±0.206 | 6.146±0.082 |
| ara | 6.747±0.124 | 6.863±0.192 | 6.92±0.072 |
| mannanase | 6.568±0.072 | 6.748±0.184 | 6.727±0.06 |
| xyla | 7.056±0.096 | 6.863±0.211 | 7.103±0.072 |
| xylanase | 6.732±0.114 | 6.811±0.173 | 6.846±0.071 |
| cdh | 7.337±0.078 | 7.631±0.192 | 7.425±0.09 |
| cellobiase | 6.454±0.131 | 6.368±0.198 | 6.622±0.075 |
| endoglucanase | 6.983±0.106 | 6.946±0.178 | 7.001±0.082 |
| pectinase (pectate_lyase) | 6.234±0.076 | 6.644±0.218 | 6.528±0.075 |
| acetylglucosaminidase | 6.479±0.13 | 6.541±0.223 | 6.646±0.078 |
| endochitinase | 6.324±0.112 | 6.354±0.187 | 6.435±0.071 |
| exochitinase | 5.832±0.229 | 5.839±0.246 | 6.088±0.096 |
| assa | **6.223±0.259a** | **5.502±0.102b** | **6.642±0.193a** |
| camdcab | **9.058±0.148a** | **8.435±0.323b** | **9.137±0.122a** |
| limeh | 7.311±0.09 | 7.29±0.248 | 7.446±0.066 |
| lmo | 7.754±0.236 | 7.649±0.229 | 8.122±0.141 |
| vana | 7.517±0.098 | 7.385±0.186 | 7.457±0.044 |
| vdh | 7.008±0.178 | 6.949±0.191 | 7.129±0.029 |
| glx | **5.565±0.639b** | **6.383±0.276a** | **6.589±0.172b** |
| lipase_fungi | 5.143±0.04 | 6.322±0.41 | 5.242±0.046 |
| mnp | 7.48±0.105 | 7.249±0.204 | 7.474±0.111 |
| phenol_oxidase | 5.693±0.645 | 6.435±0.247 | 6.515±0.096 |
| **Nitrogen cycling** | |  |  |
| amoA | 6.284±0.148 | 6.828±0.277 | 6.612±0.148 |
| hao | 5.27±0.128 | 5.85±0.153 | 5.621±0.147 |
| narG | 7.401±0.084 | 7.312±0.176 | 7.436±0.076 |
| nirS | 6.432±0.11 | 6.52±0.253 | 6.583±0.122 |
| nirK | 6.503±0.119 | 6.483±0.26 | 6.608±0.096 |
| norB | 7.068±0.098 | 7.07±0.224 | 7.188±0.113 |
| nosZ | 6.836±0.101 | 6.754±0.213 | 6.849±0.091 |
| nifH | 6.189±0.131 | 6.281±0.246 | 6.318±0.091 |
| napA | 6.46±0.126 | 6.485±0.255 | 6.43±0.086 |
| nrfA | 6.387±0.125 | 6.546±0.252 | 6.934±0.093 |
| nirB | 6.963±0.098 | 6.871±0.235 | 7.164±0.059 |
| nirA | 7.265±0.079 | 7.172±0.199 | 7.235±0.155 |
| nasA | 6.761±0.15 | 6.624±0.242 | 6.98±0.08 |
| gdh | 7.062±0.148 | 6.786±0.208 | 7.256±0.092 |
| ureC | 7.035±0.106 | 7.052±0.195 | 7.195±0.076 |

^a^WLM: soil located in Shanxi Province, temperate zone; FNM: soil located in Henan Province, the boundary between temperate and subtropical zones; SNJ: soil located in Hubei Province, subtropical zone.

^b^Values in the table are the mean values of ten replicates. Letters behind the values indicate significant differences (*P* < 0.050) among different sites, as determined by one-way analysis of variance in the SPSS. If one site shares a letter with another site, the difference between these two sites is insignificant (*P* > 0.050)

**Table S4** Topology properties of networks and random networks. To generate reliable correlations, we removed OTUs or functional genes detected in less than nine out of ten replicates collected in each mountain. As quantitative measurement of abundant genes was more accurate than those of rare genes, we used the most abundant 3,000 genes associated with carbon cycling for network reconstruction.

| **Networks** | **OTUs** | | | **Carbon cycling** | | | **Nitrogen cycling** | | |
| --- | --- | --- | --- | --- | --- | --- | --- | --- | --- |
|  | **WLM** | **FNM** | **SNJ** | **WLM** | **FNM** | **SNJ** | **WLM** | **FNM** | **SNJ** |
| number of original genes^a^ | 1125 | 759 | 684 | 3000 | 3000 | 3000 | 2300 | 1956 | 2478 |
| similarity threshold | 0.92 | 0.92 | 0.92 | 0.97 | 0.97 | 0.97 | 0.94 | 0.94 | 0.94 |
| network size(n)^b^ | 264 | 314 | 255 | 547 | 1574 | 497 | 972 | 1509 | 961 |
| R square of scale free | 0.97 | 0.91 | 0.92 | 0.76 | 0.86 | 0.91 | 0.83 | 0.77 | 0.91 |
| Modulariy | 0.94 | 0.55 | 0.66 | 0.57 | 0.64 | 0.96 | 0.81 | 0.32 | 0.82 |
| Avg connectivity(avgK) | 1.42 | 5.1 | 3.44 | 5.56 | 11.02 | 1.58 | 4.13 | 46.31 | 2.93 |
| Avg clustering coefficient (avgCC) | 0.07 | 0.23 | 0.21 | 0.22 | 0.3 | 0.1 | 0.21 | 0.43 | 0.18 |
| Avg path length (GD)^c^ | 0.34 | 2.23 | 2 | 0.16 | 3.44 | 0.04 | 2.27 | 4.41 | 2.28 |
| Transitivity (Trans) | 0.22 | 0.41 | 0.33 | 0.62 | 0.45 | 0.36 | 0.61 | 0.54 | 0.34 |
| Percentage of positive links (%) | 60.4 | 76.1 | 72.9 | 91.4 | 94.1 | 80.1 | 89.1 | 90.6 | 81.6 |
| **Random networks** |  |  |  |  |  |  |  |  |  |
| Avg clustering coefficient (avgCC) | 0.004 ± 0.002 | 0.050 ± 0.008 | 0.027 ± 0.007 | 0.073 ± 0.006 | 0.034 ± 0.002 | 0.003 ± 0.002 | 0.026 ± 0.003 | 0.194 ± 0.004 | 0.005 ± 0.002 |
| Avg path length (GD)^c^ | 0.187 ± 0.130 | 3.153 ± 0.140 | 3.417 ± 0.200 | 2.802 ± 0.115 | 3.192 ± 0.043 | 1.446 ± 0.456 | 3.360 ± 0.106 | 2.572 ± 0.020 | 4.043±0.144 |
| Transitivity (Trans) | 0.004 ± 0.007 | 0.087 ± 0.006 | 0.049 ± 0.008 | 0.177 ± 0.007 | 0.055 ± 0.001 | 0.005 ± 0.006 | 0.062 ± 0.003 | 0.248 ± 0.001 | 0.011 ± 0.002 |

^a^The number of genes originally used for network construction using the RMT-based algorithm.

^b^The number of genes (i.e., nodes) in a network.

^c^GD, geodesic distance.

**Table S5** *R* values of correlations between phyla and functional dissimilarities in three forests.

| Phylum | Correlations with functional dissimilarity | | |
| --- | --- | --- | --- |
|  | WLM | FNM | SNJ |
| *Verrucomicrobia* | 0.206 | 0.725 | 0.206 |
| *Planctomycetes* | 0.048 | 0.622 | 0.046 |
| *Actinobacteria* | 0.280 | 0.603 | 0.217 |
| *Acidobacteria* | 0.233 | 0.592 | 0.110 |
| *Proteobacteria* | 0.234 | 0.579 | 0.110 |
| *Unclassified* | 0.282 | 0.550 | 0.214 |
| *Crenarchaeoa* | 0.348 | 0.544 | 0.367 |
| *Firmicutes* | 0.199 | 0.497 | 0.035 |
| *Bacteriadetes* | 0.197 | 0.455 | 0.334 |
| *Nitrospira* | 0.003 | 0.359 | 0.293 |
| *Chloroflexi* | 0.010 | 0.334 | 0.046 |
| *Armatimoadetes* | 0.142 | 0.233 | 0.017 |
| *Gemmaproteobacteria* | 0.170 | 0.220 | 0.135 |
| *WS3* | 0.151 | 0.141 | 0.410 |
| *OD1* | 0.005 | 0.073 | 0.079 |
| *Chlamydiae* | 0.222 | 0.071 | 0.113 |

**Table S6** *R* values of correlations between functional gene categories and *Verrucomicrobia* taxonomic dissimilarities in three forests.

| Gene Category | Correlation with *Verrucomicrobia* dissimilarity | | |
| --- | --- | --- | --- |
|  | WLM | FNM | SNJ |
| starch | 0.196 | 0.645 | 0.244 |
| hemicellulose | 0.090 | 0.635 | 0.178 |
| cellulose | 0.227 | 0.730 | 0.196 |
| pectin | 0.122 | 0.718 | 0.191 |
| chitin | 0.191 | 0.570 | 0.200 |
| aromatic | 0.229 | 0.732 | 0.193 |
| lignin | 0.175 | 0.716 | 0.135 |
|  |  |  |  |
| ammonification | 0.202 | 0.747 | 0.311 |
| annamox | 0.107 | 0.554 | 0.084 |
| nitrogen assimilation | 0.040 | 0.468 | 0.238 |
| assimilatory nitrogen reduction | 0.232 | 0.678 | 0.273 |
| denitrification | 0.270 | 0.693 | 0.114 |
| dissimilatory nitrogen reduction | 0.230 | 0.771 | 0.044 |
| nitrification | 0.148 | 0.625 | 0.191 |
| nitrogen fixation | 0.165 | 0.779 | 0.157 |

## Figure list:

**Fig. S1** Detrended correspondence analysis (DCA) of microbial community based on (A) high-throughput sequencing data and (B) GeoChip data. The values for Axes 1 and 2 are percentages of variation attributed to the corresponding axis.

**Fig. S2** Heatmap showing the total relative abundances of the most unevenly distributed and most abundant OTUs in three forests.

**Fig. S3** Comparison of the positive (left panel) and negative (right panel) linkages of the association networks constructed from the WLM, FNM and SNJ forests.

**Fig. S4** Comparison of the taxonomic distribution of the most important phyla among the WLM, FNM and SNJ forests.

**Fig. S5** Average relative abundances of different phyla, normalized to the abundance of every probe. All data are presented as mean ± s.e. calculated from biological triplicated. Significant (P < 0.050) difference among sites are indicated by alphabetic letters above the bars.


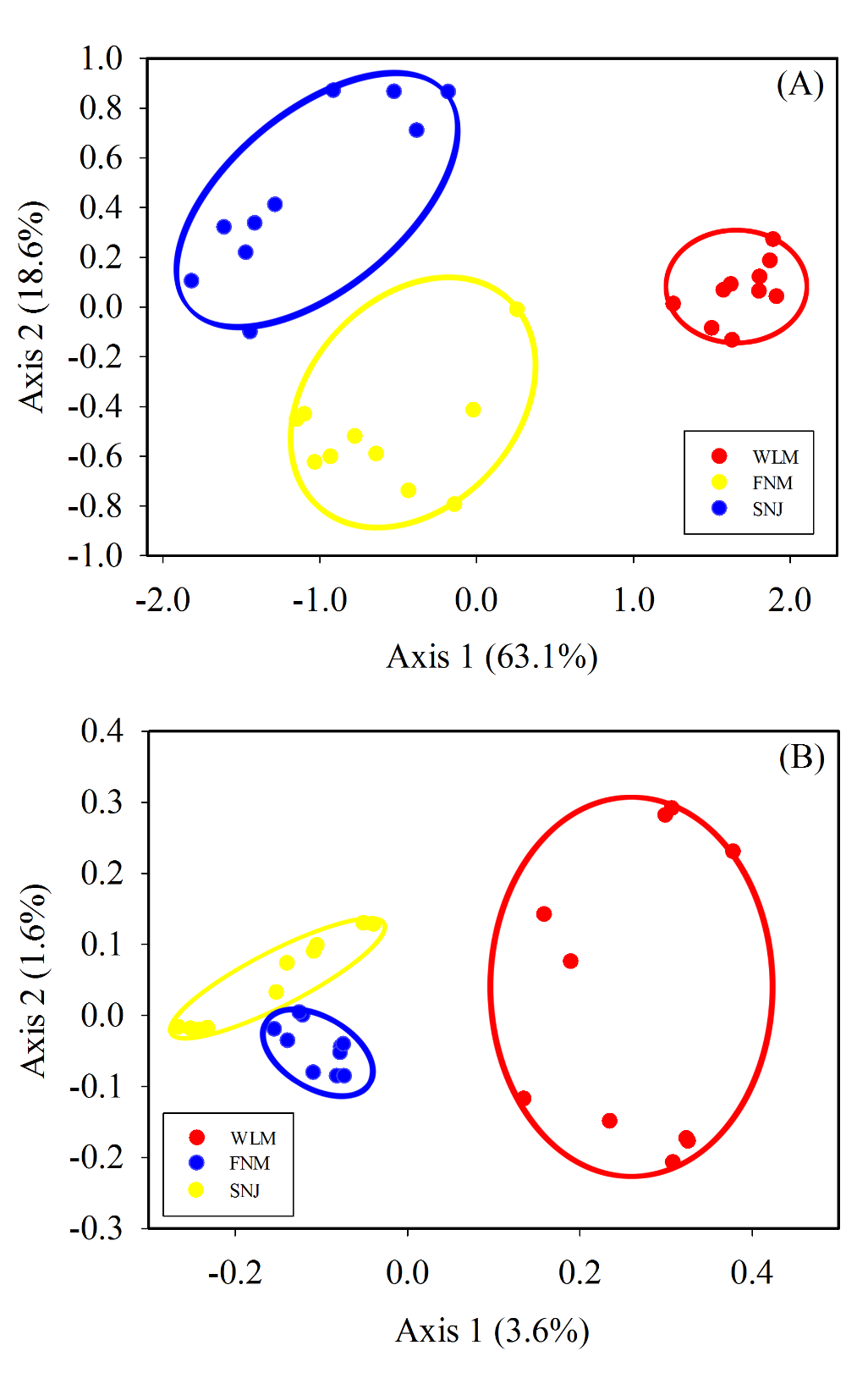


**Fig. S1** Detrended correspondence analysis (DCA) of microbial community based on (A) high-throughput sequencing data and (B) GeoChip data. The values for Axes 1 and 2 are percentages of variation attributed to the corresponding axis.


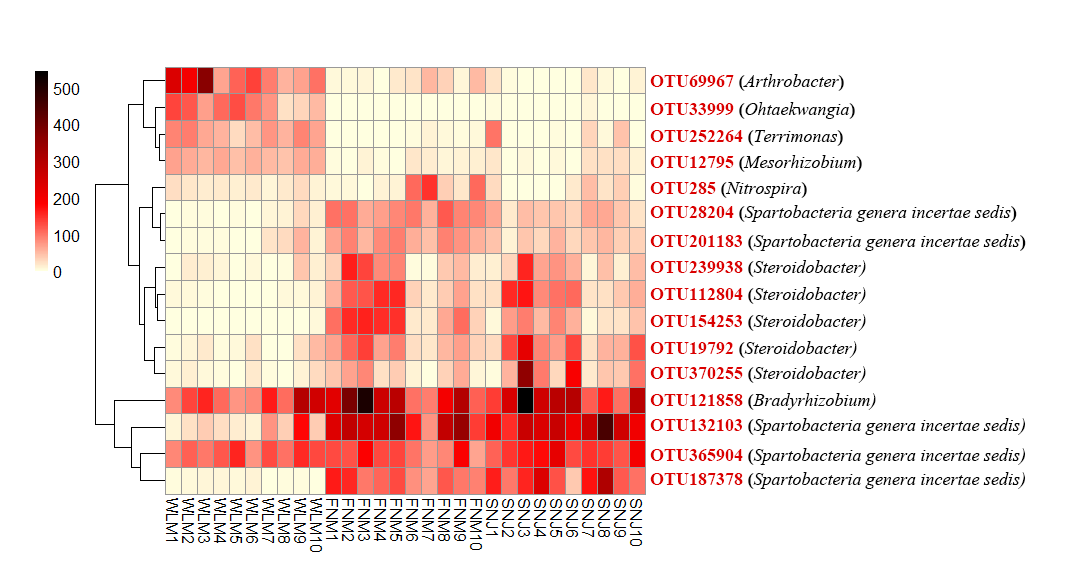


**Fig. S2** Heatmap showing the total relative abundances of the most unevenly distributed and most abundant OTUs in three forests.


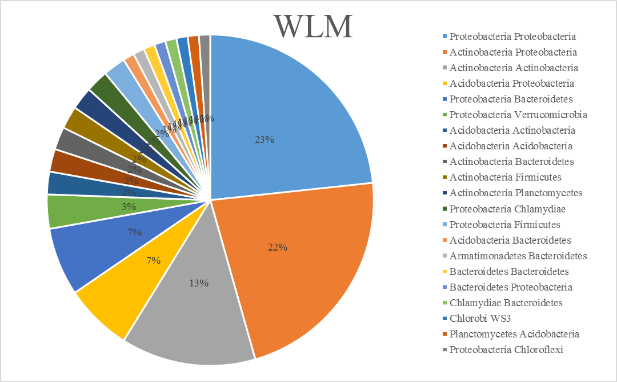

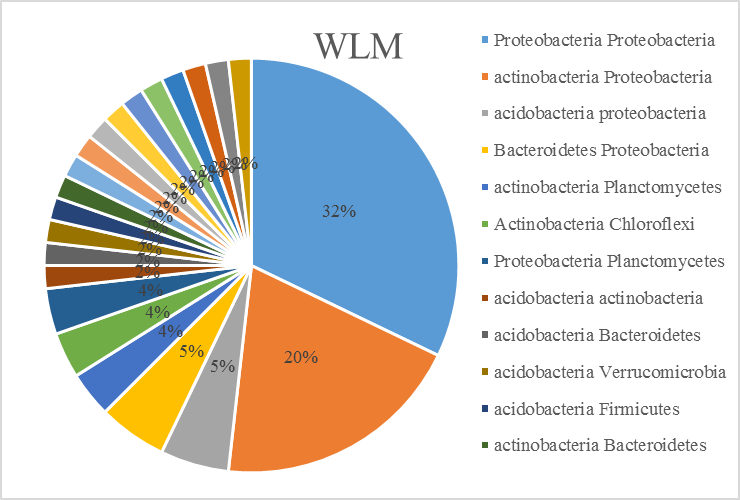


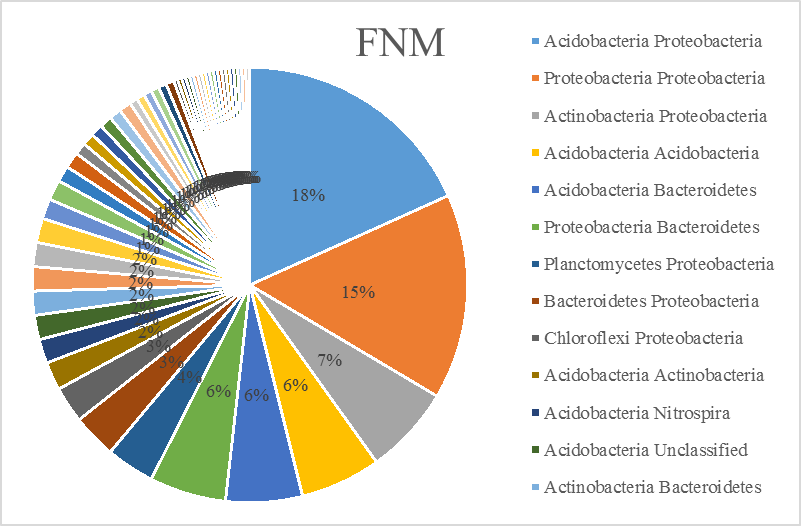

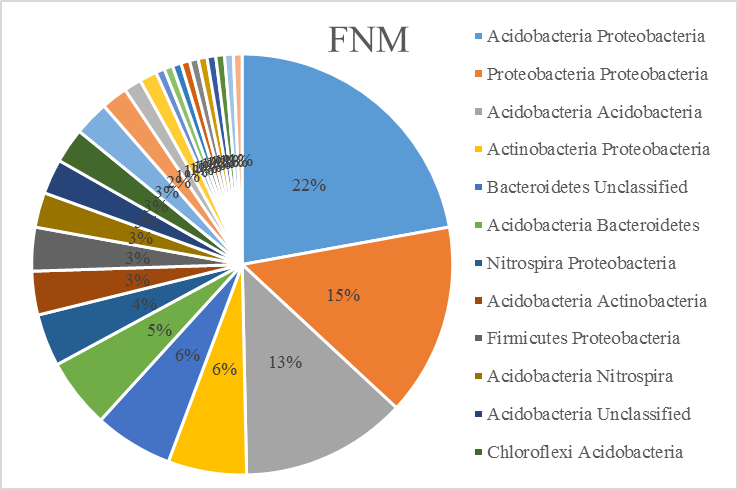


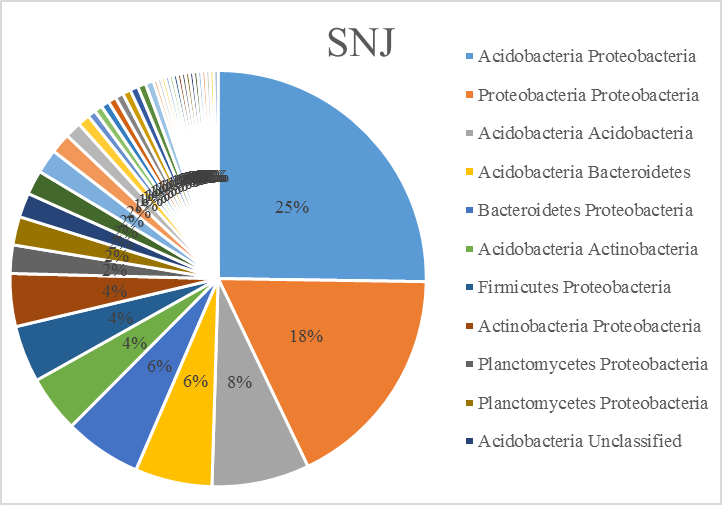

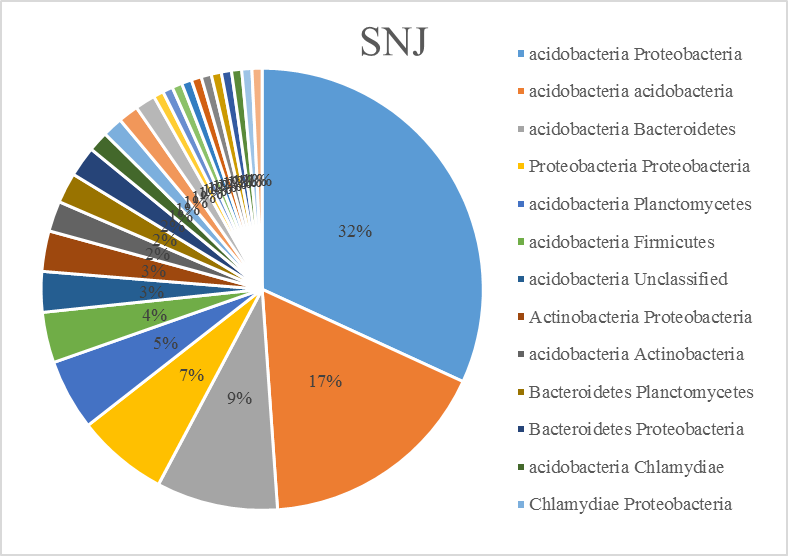


**Fig. S3** Comparison of the positive (left panel) and negative (right panel) linkages of the association networks constructed from the WLM, FNM and SNJ forests.

**

**

**Fig. S4** The correlations between plant and microbial taxonomic α-diversity in WLM, FNM and SNJ samples. Shannon indexes were calculated and plotted to generate Pearson correlation values.

**
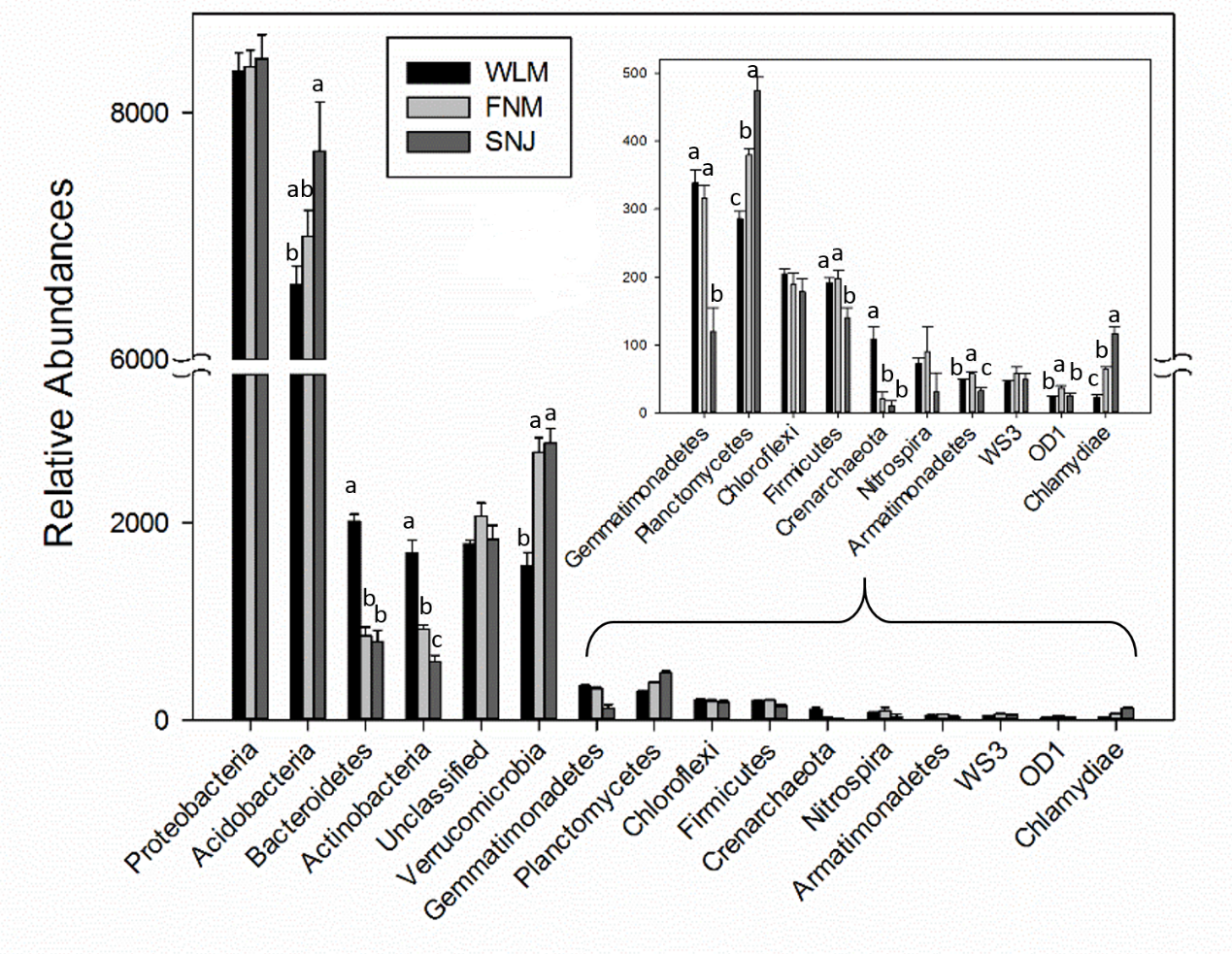
**

**Fig. S5** Relative abundances of different phyla. All data are presented as mean ± s.e. calculated from biological triplicates. Significant (*P* < 0.050) difference among sites are indicated by alphabetic letters above the bars.
